# Tumor immunity is related to ^18^F‐FDG uptake in thymic epithelial tumor

**DOI:** 10.1002/cam4.4176

**Published:** 2021-08-07

**Authors:** Hisao Imai, Kyoichi Kaira, Kosuke Hashimoto, Hiroyuki Nitanda, Ryo Taguchi, Akitoshi Yanagihara, Tetsuya Umesaki, Ou Yamaguchi, Atsuto Mouri, Tomonori Kawasaki, Masanori Yasuda, Kunihiko Kobayashi, Hirozo Sakaguchi, Ichiei Kuji, Hiroshi Kagamu

**Affiliations:** ^1^ Department of Respiratory Medicine Comprehensive Cancer Center International Medical Center Saitama University Hospital Hidaka‐City Saitama Japan; ^2^ Department of Pathology Comprehensive Cancer Center International Medical Center Saitama University Hospital Hidaka‐City Saitama Japan; ^3^ Department of General Thoracic Surgery Comprehensive Cancer Center International Medical Center Saitama University Hospital Hidaka‐City Saitama Japan; ^4^ Department of Nuclear Medicine Comprehensive Cancer Center International Medical Center Saitama University Hospital Hidaka‐City Saitama Japan

**Keywords:** ^18^F‐FDG uptake, GLUT1, HIF‐1α, immunohistochemistry, PD‐L1, PD‐L2, thymic epithelial tumor

## Abstract

**Background:**

2‐deoxy‐2‐[fluorine‐18] fluoro‐d‐glucose (^18^F‐FDG) positron emission tomography (^18^F‐FDG‐PET) is a convenient modality to assess the metabolic activity within tumor cells. However, there is no consensus regarding the relationship between ^18^F‐FDG uptake and the immune environment in thymic epithelial tumors (TETs). We conducted a clinicopathological study to elucidate the relationship between ^18^F‐FDG uptake and programmed death ligands 1 and 2 (PD‐L1/PD‐L2) expression in patients with TETs. Methods: A total of 108 patients with histologically confirmed TETs classified as thymomas or thymic carcinomas who underwent surgical resection or biopsy or needle biopsy and ^18^F‐FDG PET before any treatment between August 2007 and March 2020 were enrolled in this study. Tumor specimens underwent immunohistochemical staining for PD‐L1, PD‐L2, GLUT1, HIF‐1α, VEGFR2, VEGF‐C, and β2 adrenergic receptor. Results: High uptakes of SUV_max_, SUV_mean_, MTV, and TLG were identified in 28 (25.9%), 61 (56.5%), 55 (50.9%), and 55 (50.9%) of 108 patients, respectively. High uptake of SUV_max_ significantly correlated with PS (performance status) of 1–2, thymic carcinoma, and advanced stage, and SUV_max_ on ^18^F‐FDG uptake displayed a close association with PD‐L1 and PD‐L2 expressions, but not with MTV and TLG. Our analysis revealed that SUV_max_ was identified as being significant relationship for positive PD‐L1/PD‐L2 expression. GLUT1, HIF‐1α, and VEGFR2 were significantly associated with the expression of PD‐L1/PD‐L2 from the biological viewpoint.

**Conclusion:**

^18^F‐FDG accumulation was closely associated with the expression of PD‐L1/PD‐L2, which, in turn, was correlated with glucose metabolism and hypoxia. PD‐L1/PD‐L2 could affect the glucose metabolism and hypoxia in thymic tumor cells.

## Graphical abstract

High expression of PD‐L1/PD‐L2 was closely associated with high accumulation of 18F‐FDG.　Angiogenetic markers were linked to the expression of PD‐L1/PD‐L2.　PD‐L1 and PD‐L2 exhibited a close relationship with upregulation of tumor glucose metabolism and hypoxia.

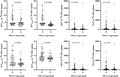

## 1 BACKGROUND

Thymic epithelial tumors (TETs), which are generally classified as thymomas and thymic carcinomas, are uncommon neoplasms present in less than 2.0% of all malignancies.^1^ In particular, thymic carcinoma is a rare cancer with a dismal outcome and no available therapeutic agents for its advanced form. Thus, the identification of new targets that can serve as predictive and prognostic markers for the development of an optimal treatment plan is essential.

2‐deoxy‐2‐[fluorine‐18] fluoro‐d‐glucose (^18^F‐FDG) positron emission tomography (^18^F‐FDG‐PET) is a convenient modality to assess the metabolic activity within tumor cells, although it shows some limitations such as false‐positive findings.^2^ Although it has been already known as one of the main biological mechanisms, glucose metabolism, hypoxia, and angiogenesis are closely linked to the accumulation of ^18^F‐FDG within tumor cells. In particular, several studies have demonstrated that the expression levels of glucose transporter 1 (GLUT1) and hypoxia‐inducible factor‐1α (HIF‐1α) are correlated with ^18^F‐FDG uptake in thoracic tumors.^3^ The ^18^F‐FDG uptake level can help to predict the grade of malignancy in TETs, allowing staging of the extent of the disease, prognosis, and therapeutic sensitivity.^3^ Programmed death ligand‐1 (PD‐L1) has been recently shown to be expressed in patients with TETs and is closely correlated with the grade of malignancy and survival.^4^, ^5^ Immune checkpoint inhibitors (ICIs) targeting programmed death‐1 (PD‐1) or PD‐L1 have been identified as effective therapeutic agents for patients with various human cancers. In particular, PD‐L1 expression within tumor cells is thought to be a predictor of response to and outcome of therapy in patients with advanced lung cancer who received anti‐PD‐1 antibody.^6^ Therefore, ICIs could serve as a potential optimal treatment option for neoplasms with PD‐L1 expression.

Several recent studies have shown that PD‐L1 expression within tumor cells is closely related to ^18^F‐FDG uptake.^7^, ^8^, ^9^, ^10^ In patients with non‐small cell lung cancer (NSCLC), PD‐L1 expression is linked to ^18^F‐FDG uptake, GLUT1, and HIF‐1α. Also, GLUT1 and HIF‐1α have been described to be closely associated with angiogenesis such as vascular endothelial growth factor (VEGF).^2^ A recent investigation indicated that the increased expression of HIF‐1α is associated with enhanced expression of PD‐L1, and contributes to the activation of T‐cell function and mitogen‐activated protein kinase (MAPK) and phosphoinositide 3‐kinase (PI3K) signaling pathways.^11^ Furthermore, HIF‐1α directly binds to the hypoxia response element in the proximal promoter of PD‐L1 and controls its expression under hypoxia.^12^ Thus, our hypothesis is that the percentage of glucose metabolism determined by HIF‐1α is suggestive of an immune reaction according to PD‐L1 expression. However, little is known about the relationship between ^18^F‐FDG uptake and PD‐L1 expression in patients with TETs. Moreover, anti‐PD‐1 antibody has been already known to provide an optimal blockade of PD‐L1 and PD‐L2, and some reports have shown that the expression of PD‐L2 may be a potential prognostic marker in lung cancer.^13^, ^14^ Nevertheless, it remains unclear whether PD‐L2 expression is associated with ^18^F‐FDG uptake and tumor aggressiveness in patients with TETs. Although maximal standardized uptake value (SUV_max_) has been generally used as a measurement of ^18^F‐FDG uptake, it remains unknown about the correlation between PD‐L1 expression and metabolic tumor volume (MTV) or total lesion glycolysis (TLG) on ^18^F‐FDG uptake. Thus, not only SUV_max_ but also MTV or TLG should be investigated for the association of PD‐L1 expression with ^18^F‐FDG uptake.

To address this gap in the literature, we conducted a clinicopathological study to elucidate the relationship between ^18^F‐FDG uptake and PD‐L1/PD‐L2 expression in patients with TETs and correlated the findings with GLUT1 and HIF‐1α expression.

## 2 MATERIALS AND METHODS

### 2.1 Patients

A total of 118 consecutive patients with histologically confirmed TETs classified as thymomas or thymic carcinomas who underwent surgical resection or biopsy or needle biopsy and ^18^F‐FDG PET before any treatment at our institution between August 2007 and March 2020 were enrolled in this study. Of them, 10 patients were excluded because of inadequate tumor specimens and radiographic information, therefore, a total of 108 patients were enrolled in this study. Pathological diagnosis and tumor subtyping were performed according to the 2015 WHO histological classification of TETs and the TNM staging system.^15^ The diagnoses were confirmed using light microscopy and immunohistochemistry. Surgically resected or biopsied primary tumors (*n* = 108) were included in this study in accordance with the institutional guidelines and the Helsinki Declaration. Ninety‐four patients received surgical resection, and biopsy was performed in 14 patients. This study was approved by the institutional ethics committee. The requirement for written informed consent was waived by the ethics committee of our institution because of the retrospective nature of the study.

### 2.2 Immunohistochemical staining

For PD‐L1 and PD‐L2, immunohistochemical staining was performed according to previously described procedures.^8^, ^9^ Rabbit monoclonal antibodies against PD‐L1 (clone 28–8; 1:100 dilution; Cell Signaling Technology, Danvers, MA, USA) and a mouse monoclonal antibody against PD‐L2 (clone 366C.9E5; 1:100 dilution; Merck KGaA) were used. Antigen retrieval was performed by autoclaving using Target Retrieval Solution (AR6, 10× Universal HIER antigen retrieval reagent; Abcam), and the reaction was visualized using Signal Stain Boost IHC Detection Reagent. The expression of PD‐L1 and PD‐L2 was considered positive when membranous staining was observed. The following semiquantitative scoring method was used for PD‐L1 and PD‐L2: 1 = <1%, 2 = 1%–24%, 3 = 25%–49%, and 4 = >50% positively stained cells.^8^, ^9^ Tumors with a score ≥2 were graded as showing positive expression.

The expressions of GLUT1 (1:100 dilution; Abcam), HIF‐1α (1:100 dilution; Abcam), vascular endothelial growth factor receptor 2 (VEGFR2) (1:100 dilution; Abcam), VEGF‐C (1:50 dilution; Immuno‐Biological Laboratories Co., Ltd.), and β2 adrenergic receptor (β2‐AR) (1:100 dilution; Abcam) were scored according to the stained tumor areas as follows: 1 = ≤10% staining, 2 = 11%–24% staining, 3 = 25%–49% staining, and 4 = ≥50% staining.^3^ Low and high expressions were defined by scores of 1–2 and 3–4, respectively, for GLUT1, HIF‐1α, and VEGFR2, and positive and negative expressions were defined by scores of 1 and 2–4, respectively, for VEGF‐C and β2‐AR.^3^

Sections were evaluated using a light microscope in a blinded fashion by at least two authors. In case of discrepancies, both investigators evaluated the slides simultaneously until they reached a final consensus on the assessment. The investigators were blinded to the patient outcomes.

### 2.3 PET imaging and data analysis

Patients fasted for at least 6 h before PET imaging, which was performed using a PET/CT scanner (Biograph 6 or 16, Siemens Healthineers K.K.) with a 585‐mm field of view. Three‐dimensional data acquisition was initiated for 60 min after injecting 3.7 MBq/kg of FDG. We acquired eight bed positions (2‐min acquisition per bed position) according to the range of imaging. Attenuation‐corrected transverse images obtained with ^18^F‐FDG were reconstructed with the ordered subset expectation maximization algorithm, based on the point spread function into 168 × 168 matrices with a slice thickness of 2.00 mm.

For the semiquantitative analysis, functional images of the standardized uptake value (SUV) were produced using attenuation‐corrected transaxial images with the injected dosage of ^18^F‐FDG, patient's body weight, and the cross‐calibration factor between PET and the dose calibrator. The SUV was defined as follows:

$$\begin{array}{cc} \text{SUV}= & \text{Radioactive concentration in the volume of interest}\left(\text{VOI}\right)\left(\text{MBq}/g\right)\\ & /\text{Injected dose}\left(\text{MBq}\right)/{\text{Patient}}^{\prime }\text{s body weight}\left(g\right). \end{array}$$

CT scanning for initial staging was performed with intravenous contrast medium, and the CT images were interpreted by board‐certified radiologists. We used RAVAT software (Nihon Medi‐physics Co. Ltd.) on a Windows workstation to semi‐automatically calculate the maximum of SUV (SUV_max_) and metabolic tumor volume (MTV), total lesion glycolysis (TLG), defined as MTV multiplied by SUV_mean_, of each lesion using SUV thresholds obtained by the SUV in the liver VOI. Each threshold was defined as average of SUV (SUV_mean_) plus 1.5×S.D. of SUV in the liver. These SUV thresholds were the optimum values to generate a 3D volume of interest (VOI) in which the whole tumor mass is completely enclosed in all cases, with CT image as the reference. In case of the activity other than tumors, including myocardium, gastro‐intestinal tracts, kidneys, and urinary tracts, were eliminated by manually according to the diagnosis by the board‐certified nuclear medicine physician.

### 2.4 Statistical analysis

Statistical analyses were performed using Student's *t*‐test, and the *χ* ^2^ test was performed for continuous and categorical variables, respectively. A *p* value < 0.05 was considered to be statistically significant. Univariate and multivariate analyses of the relationship between PD‐L1 expression and different variables were performed by logistic regression analysis. Receiver operating characteristic (ROC) curve analyses were used to evaluate the potential for **^18^**F‐FDG uptake on PET (SUV_max_, SUV_mean_, MTV, and TLG) to discriminate high from low PD‐L1 expression, and the sensitivity and specificity were calculated to determine the optimal cut‐off value differentiating positive from negative PD‐L1 expression by the ROC curve. SUV values were used as a continuous variable and ROC analysis was performed. The correlations between SUVmax, MTV, and TLG on ^18^F‐FDG uptake were analyzed using Spearman's correlation coefficient test. All statistical analyses were performed using GraphPad Prism software (v.8.0; GraphPad Software) and JMP 14.0 (SAS Institute Inc.).

## 3 RESULTS

### 3.1 Patient characteristics and immunohistochemistry

A total of 108 patients (n_males_ = 54, n_females_ = 54; median age = 64 years; age range = 34–85 years) were enrolled in the study. Patient characteristics are listed in Table 1. A total of 49 patients (45.3%) had a smoking history, and disease stages I, II, III, and IV were recorded in 37 (34.3%), 39 (36.1%), 14 (13.0%), and 18 (16.7%) patients, respectively.

**TABLE 1:** Patient's demographics according to ^18^F‐FDG uptake using different parameters

Variables	Total	SUV_max_			SUV_mean_			MTV			TLG		
	n = 108	High n = 28	Low n = 80	*p* value	High n = 61	Low n = 47	*p* value	High n = 55	Low n = 53	*p* value	High n = 55	Low n = 53	*p* value
Age (years) ≥69/<69	76/32	21/7	55/25	0.634	48/13	18/29	**<0.001**	41/14	35/18	0.401	40/15	36/27	0.086
Gender Male / Female	54/54	19/9	35/45	**0.047**	33/28	21/26	0.437	32/23	22/31	0.085	31/24	23/30	0.248
PS (ECOG) 0 / 1–2	71/37	13/15	58/22	**0.019**	35/26	36/11	**0.042**	32/23	39/14	0.107	32/23	39/14	0.107
Smoking Yes / No	49/59	17/11	32/48	0.077	27/34	22/25	0.846	32/23	17/36	**0.007**	30/25	19/34	0.056
Histology Thymoma/Thymic ca.	81/27	8/20	73/7	**<0.001**	38/23	43/4	**<0.001**	32/23	49/4	**<0.001**	32/23	49/4	**<0.001**
Disease stage I–II / III–IV	75/33	6/22	69/11	**0.014**	37/24	38/9	**0.034**	28/25	47/6	**<0.001**	27/28	48/5	**<0.001**
PD‐L1 Positive / Negative	58/50	21/7	37/43	**0.014**	38/23	20/27	0.052	34/21	24/29	0.122	33/22	25/28	0.246
PD‐L2 Positive / Negative	61/47	22/6	39/41	**0.007**	35/26	26/21	0.847	34/21	27/26	0.331	35/20	26/27	0.174
GLUT1 High / Low	56/52	24/4	32/48	**<0.001**	38/23	18/29	**0.019**	34/21	22/31	0.053	34/21	22/31	0.053
HIF−1α High / Low	32/76	14/14	18/62	**0.008**	21/40	11/36	0.288	19/36	13/40	0.295	18/37	14/39	0.531
VEGFR2 High / Low	57/51	15/13	42/38	>0.999	28/33	29/18	0.122	29/26	28/25	>0.999	30/25	27/26	0.847
VEGF‐C Positive / Negative	59/49	9/19	50/30	**0.007**	30/31	29/18	0.243	26/29	33/20	0.127	25/30	34/19	0.056
β2‐AR Positive / Negative	35/73	16/12	19/61	**0.002**	26/35	9/38	**0.012**	21/34	14/39	0.221	19/36	16/37	0.683

Abbreviations: ^18^F‐FDG, 2‐deoxy‐2‐[fluorine‐18] fluoro‐d‐glucose; ECOG PS, Eastern Cooperative Oncology Group performance status; GLUT1, glucose transporter 1; HIF‐1α, hypoxia‐inducible factor‐1α; MTV, metabolic tumor volume; PD‐L1, programmed death ligand‐1; PD‐L2, programmed death ligand‐2; SUV_max_, maximum standardized uptake value; SUV_mean_, mean standardized uptake value; Thymic ca., thymic carcinoma; TLG, total lesion glycolysis; VEGF‐C, vascular endothelial growth factor‐C; VEGFR2, vascular endothelial growth factor receptor 2; β2‐AR, beta‐2 adrenergic receptor. Bold values mean statistically significant difference.

**FIGURE 1:**
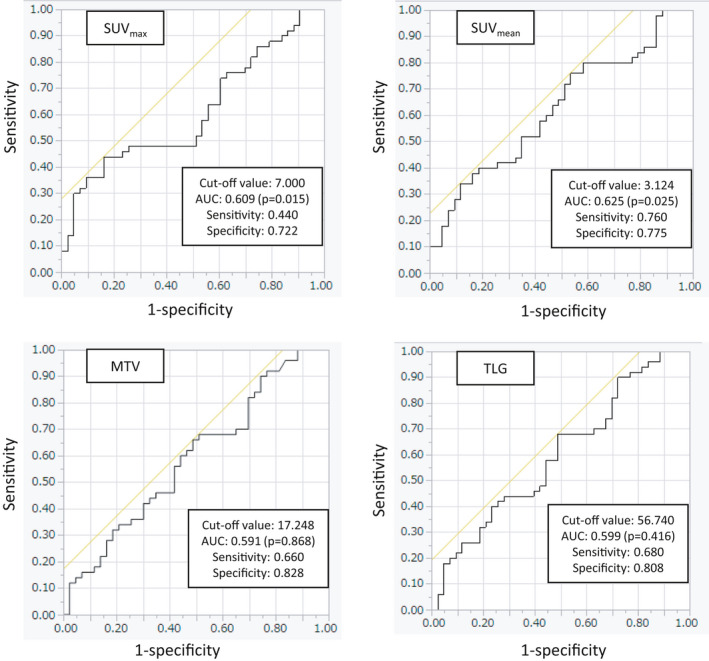
Cut‐off values for SUV_max_, SUV_mean_, MTV, and TLG were determined by receiver operating characteristic (ROC) curve analyses. Optimal ^18^F‐FDG uptake cut‐offs for SUV_max_, SUV_mean_, MTV, and TLG as determined by ROC curve analysis, were 7.0 (sensitivity: 44.0%, specificity: 72.2%, *p* = 0.015), 3.1 (sensitivity: 76.0%, specificity: 77.5%, *p* = 0.025), 17.2 cm^3^ (sensitivity: 66.0%, specificity: 82.8%, *p* = 0.868), and 56.7 gcm^3^/mL (sensitivity: 68.0%, specificity: 80.8%, *p* = 0.415), respectively

The median ^18^F‐FDG uptake values for SUV_max_, SUV_mean_, MTV, and TLG, before operation or biopsy were 4.4, 3.3, 25.5, and 93.1, respectively. The mean ± SD of SUV_max_, SUV_mean_, MTV, and TLG was 6.0 ± 3.5, 3.6 ± 1.1, 99.9 ± 226, and 403 ± 814, respectively. The optimal ^18^F‐FDG uptake cut‐offs for SUV_max_, SUV_mean_, MTV, and TLG as determined by ROC curve analysis were 7.0 (AUC: 0.609, sensitivity: 44.0%, specificity: 72.2%), 3.1 (AUC 0.625, sensitivity: 76.0%, specificity: 77.5%), 17.2 cm^3^ (AUC 0.591, sensitivity: 66.0%, specificity: 82.8%), and 56.7 gcm^3^/mL (AUC 0.599, sensitivity: 68.0%, specificity: 80.8%), respectively (Figure 1).

Representative ^18^F‐FDG PET images are shown in Figure A1 and A2 (online only). The different variables according to ^18^F‐FDG uptake by SUV_max_, SUV_mean_, MTV, and TLG were statistically compared (Table 1). The patient with more than above cut‐off values in each ^18^F‐FDG accumulation was defined as high uptake. High uptakes of SUV_max_, SUV_mean_, MTV, and TLG were identified in 28 (25.9%), 61 (56.5%), 55 (50.9%), and 55 (50.9%) patients, respectively. High uptake of SUV_max_ and SUV_mean_ was significantly correlated with PS (performance status) of 1–2, thymic carcinoma, advanced stage, and high MTV and TLG were closely associated with histology and disease stage.

### 3.2 Immunohistochemical findings

Immunohistochemical examination was performed on the 108 primary sites of the TETs. Representative images for PD‐L1, PD‐L2, GLUT1, HIF‐1α, VEGF‐C, and β2‐AR are shown in Figure A1 and A2 (online only). The immunostaining of PD‐L1 and PD‐L2 was localized predominantly in the plasma membrane of tumor cells. GLUT1 was stained on the cell membranes of tumor specimens; there was no evidence of normal tissue without red blood cells; and HIF‐1α was stained in the nuclei. The PD‐L1‐ and PD‐L2‐positive rates were 53.7% (58/108) and 56.5% (61/108), respectively, the median scores for PD‐L1 and PD‐L2 were 2.0 and 2.0, respectively, and the mean scores for PD‐L1 and PD‐L2 were 1.9 and 2.0, respectively. The percentages of scores of 1, 2, 3, and 4 for PD‐L1 and PD‐L2 were 46.3% (50/108), 26.0% (28/108), 15.7% (17/108), and 12.0% (13/108), respectively, and 43.5% (47/108), 26.9% (29/108), 13.9% (15/108), and 15.7% (17/108), respectively. The percentages of high expression and mean scores for GLUT1, HIF‐1α, and VEGFR2 were identified as 51.8% (56/108), 29.6% (32/108), and 52.7% (57/108), respectively, and 1.9, 1.5, and 1.9, respectively. The VEGF‐C and β2‐AR positive rates yielded 54.6% (59/108) and 32.4% (35/108), respectively, with the mean scores of 1.9 and 2.1, respectively. Using Spearman's correlation coefficient test, a statistically significant correlation was also observed between the expression of PD‐L1 and PD‐L2 (*ρ* = 0.27, 95% confidence interval (CI) 0.06–0.45, *p* < 0.01). Meanwhile, the expression of PD‐L1 was closely correlated with GLUT1 (*ρ* = 0.41, 95% CI 0.22–0.57, *p* < 0.01), VEGFR2 (*ρ* = 0.31, 95% CI 0.11–0.49, *p* < 0.01), and VEGF‐C (*ρ* = −0.23, 95% CI −0.41 to −0.02, *p*=0.02), but not with HIF‐1α (*ρ* = 0.18, 95% CI −0.03 to 0.37, *p*=0.08) and β2‐AR (*ρ* = 0.02, 95% CI −0.19 to 0.22, *p*=0.86), whereas, that of PD‐L2 was significantly associated with GLUT1 (*ρ* = 0.54, 95% CI 0.38–0.67, *p* < 0.01), HIF‐1α (*ρ* = 0.42, 95% CI 0.23–0.57 *p* < 0.01), VEGFR2 (*ρ* = 0.34, 95% CI 0.14–0.51, *p* < 0.01), VEGF‐C (*ρ* = −0.26, 95% CI −0.44 to −0.05, *p* = 0.01), and β2‐AR (*ρ* = 0.32, 95% CI 0.12–0.49, *p* < 0.01). Moreover, the comparison of scoring of different biomarkers according to PD‐L1 and PD‐L2 expressions was performed. Positive expression of PD‐L1 and PD‐L2 was significantly linked to the increased expression of GLUT1, HIF‐1α, and VEGFR2. But, there was opposite relationship between PD‐L1 and VEGF‐C (Figure A3, online only). The expression of PD‐L1 and PD‐L2 exhibited a significantly higher in patients with thymic carcinoma than in those with thymoma. In the analysis using Spearman's correlation coefficient, ^18^F‐FDG uptake as continuous variables was correlated with PD‐L1 and PD‐L2 expression levels. The uptake of SUV_max_ was significantly correlated with PD‐L1 (*ρ* = 0.21, 95% CI −0.01 to 0.40, *p* = 0.04) and PD‐L2 (*ρ* = 0.35, 95% CI 0.16–0.53, *p* < 0.01), and that of SUV_mean_ was significantly correlated with PD‐L1 (*ρ* = 0.22, 95% CI 0.02 –0.41, *p* = 0.02) and PD‐L2 (*ρ* = 0.34, 95% CI 0.15–0.52, *p* < 0.01). On the other hand, MTV and TLG were not identified as a significant correlation with PD‐L1 (*ρ* = 0.18, 95% CI −0.02 to 0.37, *p* = 0.07, and *ρ* = 0.20, 95% CI −0.01 to 0.39, *p* = 0.05, respectively), but, the correlation with PD‐L2 was significantly linked to MTV (*ρ* = 0.28, 95% CI 0.08–0.46, *p* < 0.01) and TLG (*ρ* = 0.32, 95% CI 0.12–0.49, *p* < 0.01).

**FIGURE 2:**
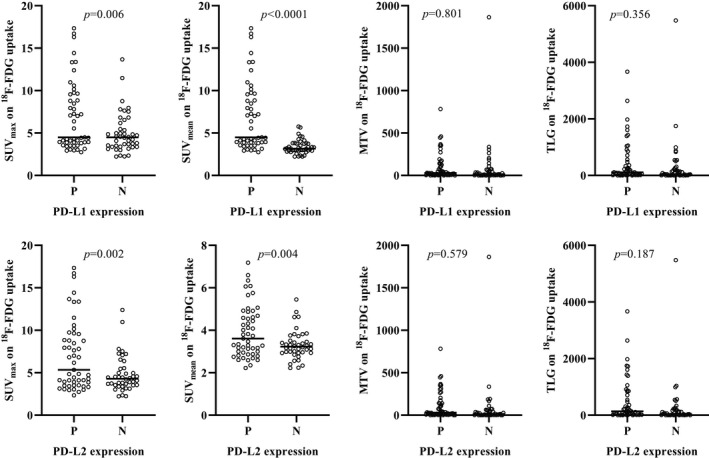
Comparison of SUV_max_, SUV_mean_, MTV, and TLG on ^18^F‐FDG uptake according to PD‐L1 and PD‐L2 expressions (B): SUV_max_ (*p* = 0.006 and *p* = 0.002) and SUV_mean_ (*p* < 0.0001 and *p* = 0.004) on ^18^F‐FDG uptake were higher in patients with positive PD‐L1 and PD‐L2 expressions than in those with negative expression. No statistically significant differences in the MTV and TLG on ^18^F‐FDG uptake were observed in patients with positive and negative PD‐L1 and PD‐L2 expressions

Figure 2 shows the comparison of SUV_max_, SUV_mean_, MTV, and TLG on ^18^F‐FDG uptake according to PD‐L1 and PD‐L2 expressions. The SUV_max_ (*p* = 0.006 and *p* = 0.002) and SUV_mean_ (*p* < 0.0001 and *p* = 0.004) on ^18^F‐FDG uptake were higher in patients with positive PD‐L1 and PD‐L2 expressions than in those with negative expression. No statistically significant differences in the MTV and TLG on ^18^F‐FDG uptake were observed in patients with positive and negative PD‐L1 and PD‐L2 expressions.

### 3.3 Univariate and multivariate analyses according to value of ^18^F‐FDG uptake on PET

Table 2 shows the univariate analysis on different variables according to the value of ^18^F‐FDG uptake. The SUV_max_, SUV_mean_, MTV, and TLG were analyzed as continuous variables of ^18^F‐FDG uptake by multiple regression analysis. Univariate analysis in the SUV_max_ demonstrated that gender, PS, smoking, disease stage, histology, PD‐L1, PD‐L2, GLIU1, HIF‐1α, VEGFR2, and β2‐AR were significant factors for predicting its uptake, but, that in the SUV_mean_, MTV, and TLG revealed that PD‐L1 and PD‐L2 were not identified as significant factors for predicting their uptakes.

**TABLE 2:** Univariate analysis on different variables according to the value of ^18^F‐FDG uptake

Variables	Different values of ^18^F‐FDG uptake (*p* value)			
	SUV_max_	SUV_mean_	MTV	TLG
Age (years) ≥69/<69	0.432	0.263	0.304	0.243
Gender Male/Female	**0.024**	0.284	**0.034**	**<0.001**
PS (ECOG) 0 / 1–2	**0.011**	0.097	**0.007**	**0.005**
Smoking Yes / No	**0.031**	0.462	0.318	0.105
Disease stage I–II / III–IV	**<0.001**	**<0.001**	**<0.001**	**<0.001**
Histology Thymoma/thymic cancer	**<0.001**	**<0.001**	**0.026**	**<0.001**
PD‐L1 Positive/Negative	**0.045**	0.286	0.869	0.414
PD‐L2 Positive/Negative	**0.036**	0.301	0.680	0.251
GLUT1 High / Low	**<0.001**	**0.011**	0.574	0.146
HIF−1α High / Low	**0.044**	0.549	0.132	**0.012**
VEGFR2 High / Low	**0.045**	0.152	0.151	0.104
VEGF‐C High / Low	0.095	0.235	0.849	0.368
β2‐AR High / Low	**0.003**	**0.026**	0.950	0.401

Abbreviations: ^18^F‐FDG, 2‐deoxy‐2‐[fluorine‐18] fluoro‐d‐glucose; 95% CI, 95% confidence interval; ECOG PS, Eastern Cooperative Oncology Group performance status; GLUT1, glucose transporter 1; HIF‐1α, hypoxia‐inducible factor‐1α; MTV, metabolic tumor volume; OR, odds ratio; PD‐L1, programmed death ligand‐1; PD‐L2, programmed death ligand‐2; SUV_mean_, mean standardized uptake value; TLG, total lesion glycolysis; UV_max_, maximum standardized uptake value; VEGF‐C, vascular endothelial growth factor‐C; VEGFR2, vascular endothelial growth factor receptor 2; β2‐AR, beta‐2 adrenergic receptor.

Bold values mean statistically significant difference.

Next, multivariate analysis was performed using different variables with significance of *p* < 0.05 on the univariate stage in the in the SUV_max_ (Table 3). By multivariate analysis, disease stage, histology, GLUT1, and HIF‐1α were identified as independent predictors for SUV_max_ on ^18^F‐FDG uptake.

**TABLE 3:** Multivariate analysis on different variables according to the value of SUV_max_

Variables	SUV_max_		
	β	95% CI	*p* value
Age (years) ≥69/<69			
Gender Male/Female	0.39	−0.225 to 1.009	0.211
PS (ECOG) 0 / 1–2	0.36	−0.206 to 0.926	0.210
Smoking Yes / No	0.14	−0.452 to 0.739	0.634
Disease stage I–II / III–IV	1.03	0.306**–**1.744	**0.005**
Histology Thymoma/thymic cancer	2.27	1.38**–**3.157	**<0.001**
PD‐L1 Positive/Negative	0.25	−0.318 to 0.828	0.379
PD‐L2 Positive/Negative	−0.38	−0.983 to 0.215	0.206
GLUT1 High / Low	0.86	0.156**–**1.566	**0.017**
HIF−1α High / Low	−0.59	−1.233 to 0.041	**0.006**
VEGFR2 High / Low	−0.95	−1.529 to −0.389	0.001
VEGF‐C High / Low			
β2‐AR High / Low	−0.27	−0.930 to 0.371	0.395

Abbreviations: ^18^F‐FDG, 2‐deoxy‐2‐[fluorine‐18] fluoro‐d‐glucose; 95% CI, 95% confidence interval; ECOG PS, Eastern Cooperative Oncology Group performance status; GLUT1, glucose transporter 1; HIF‐1α, hypoxia‐inducible factor‐1α; MTV, metabolic tumor volume; OR, odds ratio; PD‐L1, programmed death ligand‐1; PD‐L2, programmed death ligand‐2; SUV_max_, maximum standardized uptake value; SUV_mean_, mean standardized uptake value; TLG, total lesion glycolysis; VEGF‐C, vascular endothelial growth factor‐C; VEGFR2, vascular endothelial growth factor receptor 2; β2‐AR, beta‐2 adrenergic receptor. Bold values mean statistically significant difference.

## 4 DISCUSSION

To the best of our knowledge, this is the first study to evaluate the relationship between PD‐L1/PD‐L2 expression and ^18^F‐FDG uptake on PET in patients with TETs. We found that high expression of PD‐L1 and PD‐L2 was closely associated with high accumulation of ^18^F‐FDG; in particular, PD‐L1/PD‐L2 expression levels were significantly correlated with those of glucose metabolism and hypoxia. As angiogenetic markers, VEGFR2 and β2‐AR were associated with the expression of PD‐L1/PD‐L2. Moreover, we confirmed that the expression of PD‐L1 and PD‐L2 was closely associated with not MTV or TLG but SUV_max_ on ^18^F‐FDG uptake, confirmed by multivariate analysis. Overall, PD‐L1 and PD‐L2 expressions indicated a strong correlation with glucose metabolism, as determined by GLUT1. Although SUV_max_ is closely correlated with MTV and TLG on ^18^F‐FDG uptake, the upregulation of PD‐L1 and PD‐L2 may play a crucial role in the pathogenesis of tumor glucose metabolism in patients with TETs. Further studies with an experimental approach using thymic tumor cell lines are warranted to elucidate the results of our study.

Several researchers have described that PD‐L1 is frequently expressed in TETs, and a WHO classification is closely related to positive PD‐L1 expression, but there was some discrepancy regarding the trend for worsened survival.^4^, ^16^, ^17^, ^18^, ^19^ Padda et al. reported that the high expression of PD‐L1 could predict a significantly worse OS, which was correlated with more aggressive histology.^16^ However, Yokoyama et al. described that the low PD‐L1 expression and a high number of PD‐1‐positive tumor infiltrative lymphocytes (TILs) were significant predictors of worse survival in patients with thymic carcinoma.^18^ Considering the evidence from previous studies, it is debatable whether PD‐L1 could absolutely predict a worse outcome for patients with TETs. As our study also indicated that the expression of PD‐L1 was higher in thymic carcinoma than in thymoma, PD‐L1 may highly express in human neoplasms with malignant phenotype.

PD‐L1 is an important target for PD‐1 blockade, whereas PD‐L2, as another PD‐1 ligand, may also play a crucial role in the inhibition of PD‐1 in human neoplasms. The prevalence of PD‐L2 was significantly correlated with PD‐L1, and PD‐L2 status was also a significant predictor of PFS with pembrolizumab, independent of PD‐L1 status.^20^ A previous study reported that GLUT1 expression is associated with better clinical outcomes in advanced‐stage classical Hodgkin's lymphoma and is significantly associated with PD‐L1 and PD‐L2 expressions.^21^ This study supports the hypothesis that GLUT1‐related signaling pathways play an important role in the PD‐L1 or PD‐L2 pathway. Furthermore, a previous article reported that PD‐L2‐positive pheochromocytoma and paraganglioma were characterized by higher HIF‐1α expression. That study reported the enrichment of transcripts involved in the hypoxic response in relation to PD‐L2, but not PD‐L1 expression.^22^ When the researchers considered a broader subset of 200 genes involved in the hypoxic response, PD‐L2 upregulation strikingly emerged as a stronger and more substantial determinant of tumor hypoxia than PD‐L1, suggesting a potential mechanistic relationship between hypoxia and PD‐L2‐mediated antitumor immune control. Their data suggest that PD‐L2 has a more predominant role than PD‐L1 in shaping the immune‐tolerogenic environment, given the highly significant association with key pathways involved in innate, adaptive immunity, and inflammation in pheochromocytomas and paragangliomas. Recently, Rouquette et al. reported that the PD‐L2 antibody stained no tumor epithelial cells in TETs.^19^ Although we also performed PD‐L2 staining using the same antibody, no staining was observed in our study, corresponding to their results.^19^

Previous investigations have supported the potential of PD‐L1 as an alternative target of HIF‐1α and suggested that the distribution of glucose metabolism determined by HIF‐1α could reflect the immune response reflected by the expression of PD‐L1.^11^, ^12^ In addition, direct blockade of PD‐L1 within cancer cells has been reported to diminish glycolysis by inhibiting the mTOR pathway and the expression of glycolysis enzymes.^23^ Takada et al. reported the radiological features of PD‐L2 expression in 222 patients with lung adenocarcinoma.^24^ In their study, the SUV_max_ for ^18^F‐FDG uptake was found to be significantly higher in PD‐L2‐positive than in PD‐L2‐negative cases.^24^ It remains unknown why the expression level of PD‐L2 is closely related to ^18^F‐FDG uptake. PD‐L2 seemed to be more strongly correlated with glucose metabolism, hypoxia, and angiogenesis, compared with PD‐L1. Further investigation should be conducted to elucidate the relationship between PD‐L2 and ^18^F‐FDG uptake from the perspective of basic science.

Our study is a first investigation to evaluate whether MTV or TLG could be correlated with the expression of PD‐L1, thus, it remains unclear why SUV_max_ was chosen as a better marker for the close correlation of PD‐L1 expression than TLG or MTV. Considering that PD‐L1 was not identified as independent predictor for the ^18^F‐FDG uptake by SUV_max_, we feel the possibility of weak association between ^18^F‐FDG uptake and PD‐L1 expression in patients with TETs.

There are several limitations to our study. First, our study had a small sample size, which may have biased the results of our study. Since thymic cancer is a rare neoplasm, only limited numbers of samples were collected. Second, we tried to examine PD‐L1 staining using clone 28–8; however, there are several kinds of PD‐L1 clones. An additional investigation using other clones of PD‐L1 may be needed to confirm the results of our study. Moreover, the AUC for determining cut‐off value of ^18^F‐FDG uptake and Spearman correlation is relatively low, having some limitations of statistical analysis, thus, this limitation also may bias the results of our conclusion. But, there is controversial issue which cut‐off value is optimal to dichotomize the uptake value of ^18^F‐FDG on PET. Finally, the results of our study were not confirmed by experimental investigations. In the level of tumor cell lines, little is known about any data elucidating the association between PD‐L1 expression and ^18^F‐FDG uptake. Further examination is needed to approach some basic mechanism.

In conclusion, the relevance and distribution of ^18^F‐FDG uptake on PET were significantly associated with the expression of PD‐L1 and PD‐L2 in patients with TETs, and PD‐L2 seemed to be more correlated with ^18^F‐FDG uptake than PD‐L1. In particular, PD‐L1 and PD‐L2 exhibited a close relationship with upregulation of tumor glucose metabolism (GLUT1) and hypoxia (HIF‐1α), which play essential roles in the mechanism of ^18^F‐FDG uptake within tumor cells.

Further studies are needed to elucidate why PD‐L1 and PD‐L2 affect glucose metabolism and hypoxia in TETs.

## CONFLICT OF INTEREST

Kyoichi Kaira has received research grants and a speaker honorarium from Ono Pharmaceutical Company, Boehringer Ingelheim, Chugai Pharmaceutical, Taiho Pharmaceutical, Eli Lilly Japan, and AstraZeneca. Atsuto Mouri has received a speaker honorarium from Eli Lilly, Taiho Pharmaceutical, Pfizer, Chugai Pharmaceutical, and AstraZeneca. Hiroshi Kagamu has received research grants and a speaker honorarium from Ono Pharmaceutical Company, Bristol‐Myers Company, Boehringer Ingelheim, MSD, Daiichi Sankyo Company, Chugai Pharmaceutical, Taiho Pharmaceutical, Merck Biopharma Company, Eli Lilly Japan, and AstraZeneca. Kobayashi has received research grants and a speaker honorarium from Boehringer Ingelheim, AstraZeneca, and Bristol‐Myers Company.

## AUTHORS CONTRIBUTION

HI, KK, KH, and IK: Study conception and manuscript preparation. HN, RT, AY, TU, OY, and AM: Patient management. TK, MY, and KK: Statistical analysis and patient data collection. HS, IK, and HK: Manuscript revision. All authors contributed and agreed with the content of the manuscript.

## ETHICAL APPROVAL

All procedures performed in studies involving human participants were in accordance with the ethical standards of the institutional and/or national research committee and with the 1964 Helsinki declaration and its later amendments or comparable ethical standards.

## Supporting information

FigA1

FigA2

FigA3
