# Supplementary figures and images for: Tumor immunity is related to 18F‐FDG uptake in thymic epithelial tumor
Source: Cancer Med. 2021 Aug 7;10(18):6317–26. doi: 10.1002/cam4.4176 (PMC8446555; doi:10.1002/cam4.4176)

## Slide 1
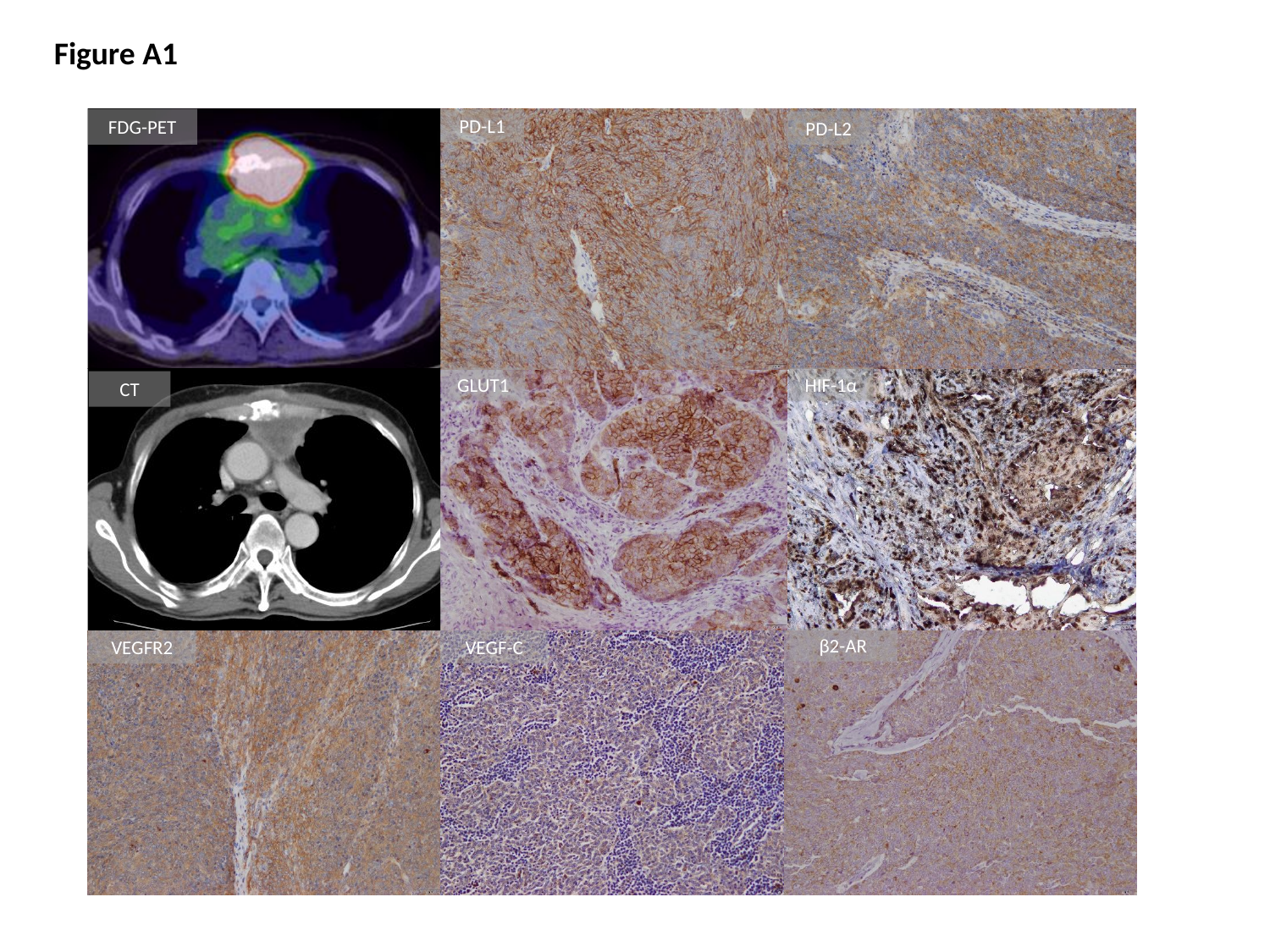

Figure A1
FDG-PET
PD-L1
PD-L2
HIF-1α
GLUT1
CT
β2-AR
VEGF-C
VEGFR2

Supplement: Supplementary file 1 — FigA1 [file CAM4-10-6317-s002.pptx]

## Slide 1
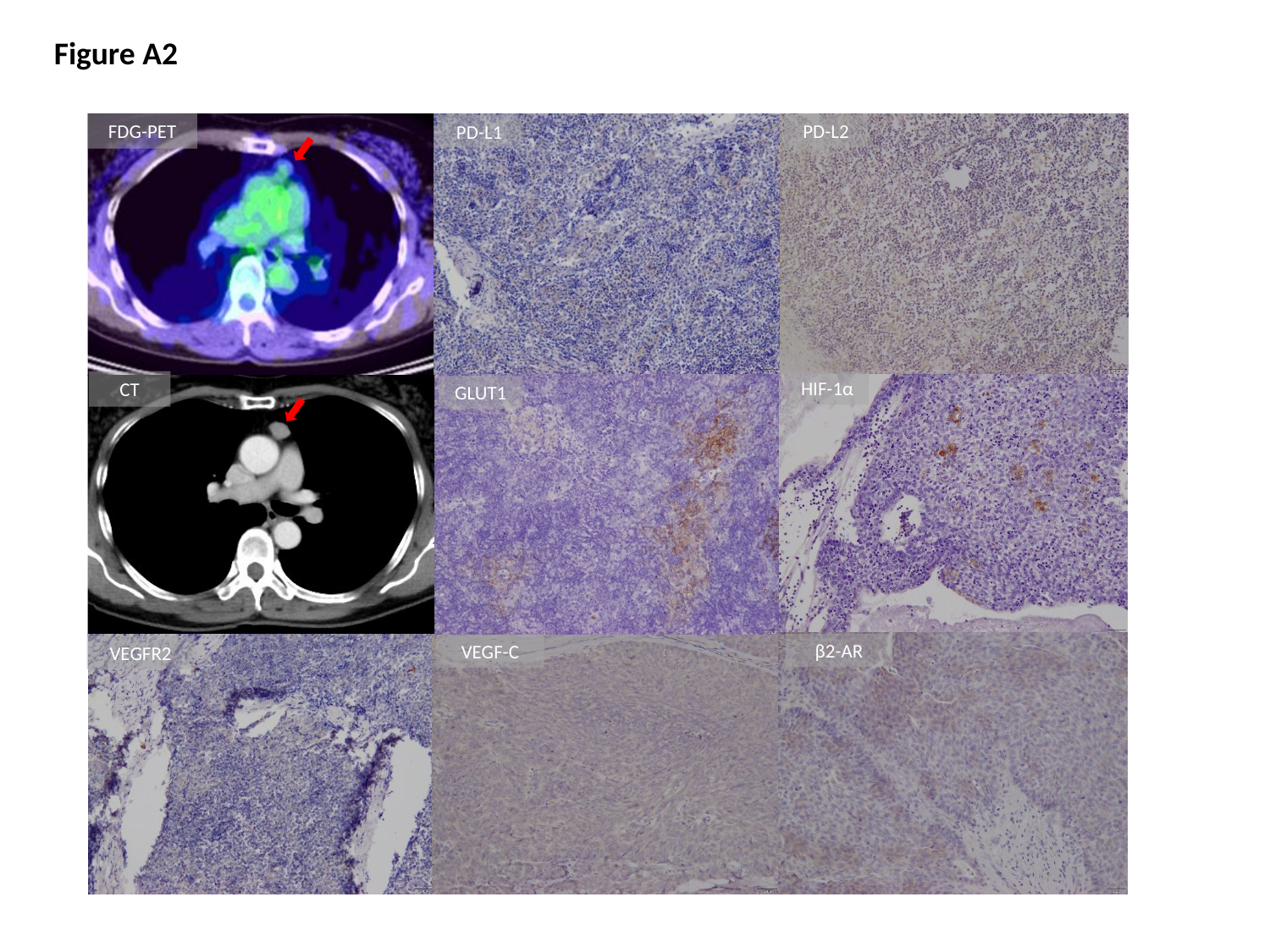

Figure A2
FDG-PET
PD-L2
PD-L1
CT
HIF-1α
GLUT1
β2-AR
VEGF-C
VEGFR2

Supplement: Supplementary file 2 — FigA2 [file CAM4-10-6317-s003.pptx]

## Slide 1
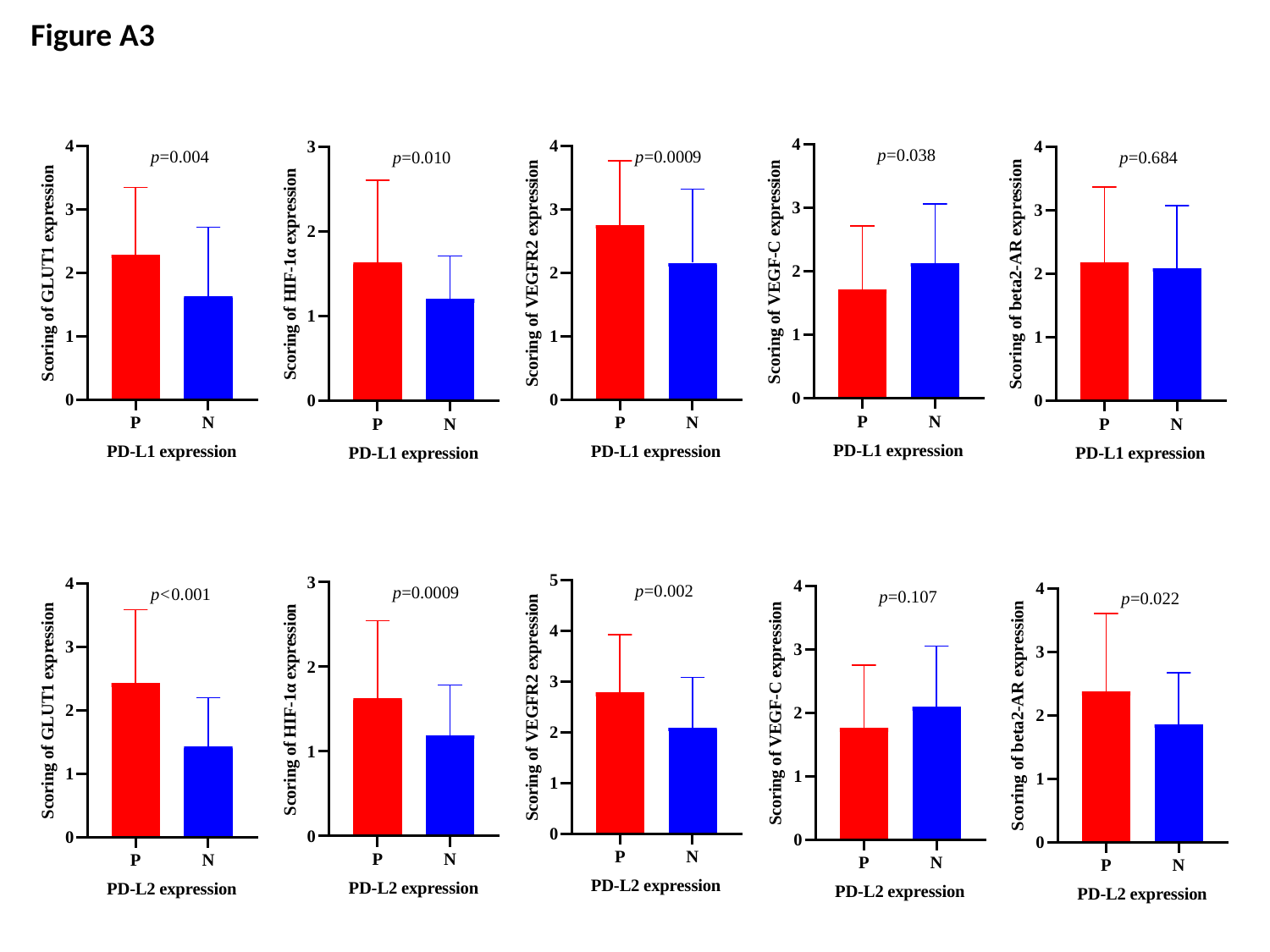

Figure A3

Supplement: Supplementary file 3 — FigA3 [file CAM4-10-6317-s001.pptx]
